# Use of Antiepileptic Drugs and Risk of Prostate Cancer: A Nationwide Case-Control Study in Prostate Cancer Data Base Sweden

**DOI:** 10.1155/2023/9527920

**Published:** 2023-02-15

**Authors:** Gincy George, Hans Garmo, Jan Adolfsson, Kristin Elf, Rolf Gedeborg, Lars Holmberg, Pär Stattin, Johan Styrke, Mieke Van Hemelrijck

**Affiliations:** ^1^Translational Oncology and Urology Research, King's College London, London, UK; ^2^Department of Surgical Sciences, Uppsala University, Uppsala, Sweden; ^3^Department of Clinical Science, Intervention and Technology, Karolinska Institutet, Stockholm, Sweden; ^4^Clinical Neurophysiology, Department of Medical Sciences, Uppsala University, Uppsala, Sweden; ^5^Department of Surgical and Perioperative Sciences, Urology and Andrology, Umeå University, Umeå, Sweden

## Abstract

An inverse association between use of antiepileptic drugs (AEDs) and prostate cancer (PCa) has been suggested, putatively due to the histone deacetylases inhibitory (HDACi) properties of the AEDs. In a case-control study in Prostate Cancer data Base Sweden (PCBaSe), PCa cases diagnosed between 2014 and 2016 were matched to five controls by year of birth and county of residence. AED prescriptions were identified in the Prescribed Drug Registry. Odds ratios (ORs) and 95% confidence intervals for risk of PCa were estimated using multivariable conditional logistic regression, adjusted for civil status, education level, Charlson comorbidity index, number of outpatient visits, and cumulative duration of hospital stay. Dose responses in different PCa risk categories and HDACi properties of specific AED substances were further explored. 1738/31591 (5.5%) cases and 9674/156802 (6.2%) controls had been exposed to AED. Overall, users of any AED had a reduced risk of PCa as compared to nonusers (OR: 0.92; 95% CI: 0.87–0.97) which was attenuated by adjustment to healthcare utilisation. A reduced risk was also observed in all models for high-risk or metastatic PCa in AED users compared to nonusers (OR: 0.89; 95% CI: 0.81–0.97). No significant findings were observed for dose response or HDACi analyses. Our findings suggest a weak inverse association between AED use and PCa risk, which was attenuated by adjustment for healthcare utilisation. Moreover, our study showed no consistent dose-response pattern and no support for a stronger reduction related to HDAC inhibition. Further studies focusing on advanced PCa and PCa treatments are needed to better analyse the association between use of AED and risk of PCa.

## 1. Background

Antiepileptic drugs (AEDs) with histone deacetylases inhibitory (HDACi) properties repress transcription in several tumour cell lines [1]. Long-term AED treatment has been suggested to reduce the risk of PCa by slowing the transformation of tumour precursor cells in the prostate [2].

Identification and repurposing of specific drugs such as AED may provide new therapeutic indications for substances with established safety profiles, potentially lower overall development costs, and shorter development timelines [3, 4]. In addition to cancer cell cycle arrest, differentiation, and cell death, HDACi also moderates angiogenesis and modifies the immune response. This renders them to be promising anticancer drugs, particularly in combination with other anticancer drugs and/or radiotherapy [5]. However, anticancer mechanisms of HDACi are not uniform, and large multicentric clinical studies are necessary to ascertain the beneficial clinical outcomes of these drugs.

Although in vitro and in vivo studies on AED have shown a clear antiproliferative effect on PCa cells, epidemiological study results have been inconsistent [2]. AED users showed reduced PCa risk compared to never-users in a case-control study based on the Finnish Cancer Registry [6]. Whilst nonsignificant risk reduction was observed for high-risk PCa, this finding was only significant for men diagnosed with low-risk PCa. Moreover, demographic factors such as age, body mass index, and the use of additional medications had no significant effect on the risk of PCa, and men on AED with or without HDACi properties showed a lower risk of developing PCa compared to never-users.

However, other studies have shown contradictory results to the Finnish study. Whereas a cohort study showed an increased risk of PCa in AED users [7], two other studies, including a cohort study and a case-control study, found no association between AED use and PCa risk [8, 9]. Since epidemiological studies are important to provide evidence and support for drug development efforts, findings on the association between AED and the risk of PCa need to be corroborated [6, 8–10]. This case-control study investigated the association between all AED and incidence of PCa.

## 2. Methods

### 2.1. Study Population

Prostate Cancer data Base Sweden (PCBaSe) is a nationwide population-based database that links the National Prostate Cancer Register (NPCR) of Sweden to other health care registries and demographic databases using the individually unique Swedish Personal Identity Number. PCBaSe 4.0 consists of more than 180,000 men on AEDs with comprehensive data on inpatient and outpatient care, prescription patterns, and socioeconomic factors [11]. Due to ethical considerations from the Swedish Board of Health and Welfare, age in PCBaSe is not captured more precisely than by year of birth and quarter of birth year.

All new PCa cases diagnosed between 2014 and 2016 were extracted from PCBaSe. The date of PCa diagnosis was used as the index date for cases and their respective controls. Each PCa case in this study was matched to five controls by their year of birth and residency. The five controls per case were selected from the general Swedish population and were free of PCa on the date of diagnosis for the corresponding case, lived in the same county, and had the same year of birth. Exposure was defined from filled prescription date for any AED in the Prescribed Drug Registry (within a fixed exposure window of 8.5 years) for all cases and controls. The AEDs included in the final analysis were sodium valproate (Anatomical Therapeutic Chemical (ATC) code: N03AG01), carbamazepine (ATC code: N03AF01), lamotrigine (ATC code: N03AX09), levetiracetam (ATC code: N03AX14), oxcarbazepine (ATC code: N03AF02), ethosuximide (ATC code: N03AD01), topiramate (ATC code: N03AX11), phenytoin (ATC code: N03AB02), gabapentin (ATC code: N03AX16), pregabalin (ATC code), clonazepam (ATC code: N03AX14), primidone (ATC code: N03AA03), phenobarbital (ATC code: N03AA02), lacosamide (ATC code: N03AX18), zonisamide (ATC code: N03AX15), and eslicarbazepine (ATC code: N03AF04). Sodium valproate, lamotrigine, levetiracetam, oxcarbazepine, and topiramate were AEDs with known HDACi properties.

### 2.2. Statistical Analysis

Odds ratios (ORs) and 95% confidence intervals (CIs) were estimated using multivariable conditional logistic regression for the risk of PCa associated with AED use. The regression models were adjusted for (i) civil status, education level, and Charlson comorbidity index (CCI) and (ii) civil status, education level, CCI, number of outpatient visits, and cumulative length of hospital stay. These variables were categorised as described in Van Hemelrijck et al. (2013) [12]. The number of outpatient visits included visits 1–10 years prior to PCa diagnosis and was categorised as follows: no visits, 1, 2, 3–5, 6–9, and ≥10 visits. The cumulative length of hospital stay was defined as days or weeks that an individual was hospitalised for, due to any reason, 1–10 years prior to study entry. This was categorised as follows: no visits, 1–3 days, 4–7 days, 1-2 weeks, 3-4 weeks, and >4 weeks. To avoid reverse causation, last year prior to PCa diagnosis was excluded for the variables number of outpatient visits and cumulative length of hospital stay.

Cumulative defined daily doses (DDDs) for AED were categorised as follows: no drug, 1–365 days DDD, 1–5 years DDD, and ≥6 years DDD. The association between this measure of cumulative dose and risk of PCa was analysed overall and separately in subcohorts defined by PCa risk categories: low- or intermediate-risk PCa and high-risk or metastatic PCa.

To determine the association between certain AED compounds with or without HDACi properties and the risk of PCa, each AED was further investigated in separate analyses.

All analyses were conducted using Software for Statistics and Data Science (STATA) version 15.

## 3. Results

Of the 31,591 cases diagnosed with PCa between 2014 and 2016 and their 156,802 controls, 1,738 cases and 9,674 controls were exposed to AED. In univariate analyses, comorbidity and healthcare utilisation were inversely associated with the risk of PCa (Table 1).

An inverse association was observed between AED and PCa in unadjusted analysis (OR: 0.89; 95% CI: 0.84–0.93; Table 1). Adjustment for civil status, education level, and CCI attenuated this association (OR: 0.92; 95% CI: 0.87–0.97; Table 2). However, this association was no longer evident when adjusted for healthcare utilisation (OR: 0.96; 95% CI: 0.91–1.02; Table 2).

When stratified by PCa risk categories, this association was only seen for men with high-risk and metastatic PCa (OR: 0.89; 95% CI: 0.81–0.97) compared to nonusers (Table 2). Moreover, there was no overall trend for cumulative dose although an inverse association was observed for men with high-risk and metastatic PCa exposed to AED with 1–5 year DDD. In the analyses of exposures to specific AED substances, no pattern suggestive of a relation between strength of association with anticipated HDACi activity or a pharmacological mechanism was observed (Table 3).

## 4. Discussion

In this population-based case-control study, there was a weak overall association between AED use and a decreased risk of PCa. However, the patterns seen in relation to cumulative AED dose, PCa risk categories, and specific AED substances do not provide consistent support for a causal association.

This study used a similar case-control approach as Salminen et al., where PCa cases were selected from the Finnish Cancer Registry and matched to controls from the Population Register Centre of Finland according to age and area of residence [6]. Even though our findings were consistent with previous observational research while using a different source population that increases the generalisability and external validity of the study results, the strength of the association was weak and further weakens when adjusted for previous healthcare utilisation. This indicates that health-seeking behaviour in men who were already on AED was largely influencing the observed results. Residual confounding that could attenuate the association further might have also influenced the results of this study.

A plausible mechanism suggested for the protective effects of AED on cancer incidence and progression is HDAC inhibition, which selectively alters gene transcription in cancer cells and leads to HDACi-induced transformed cell death [1, 13]. However, the results for individual AED substances in this study showed no evidence for the proposed mechanism of HDAC inhibition. Although an inverse association was observed with sodium valproate that has HDACi properties, this receded following adjustment for the number of outpatient visits and cumulative length of hospital stay. Moreover, no inverse association was observed between lamotrigine (another drug with HDACi properties) and the risk of PCa. The proposed beneficial effects of HDACi in PCa have also been challenged by the inconclusive results of phase I/II clinical trials [14]. Since there was no clear pattern where one specific AED would potentially select for men with reduced PCa risk in our study, it was difficult to detect any clear relation between the estimated associations observed in our study and the pharmacological mechanisms for individual AED substances, which warrants further research.

A reduced risk of high-risk or metastatic PCa was found among AED users compared to nonusers in this study. Lower risk estimates were also observed for advanced PCa in AED users compared to non-AED users in previous research [6]. This suggests potential carcinoma-suppressing actions of AED, especially in men at risk of developing more advanced disease. However, further investigation into these mechanisms in advanced PCa is required before any repurposing or reprofiling use of AED for PCa can be considered.

There was no overall trend for cumulative dose (DDD), although a weak inverse association was only observed for high-risk and metastatic PCa. A reduced risk of PCa was also observed in men with longer duration of AED use (1–5 year DDD) compared to never users. However, a larger cumulative dose of AED usage was associated with a lower risk of low and intermediate risk in previous research [6], which is not consistent with our study findings. Therefore, the dose-response pattern in the current study does not provide clear support for a causal effect, and the observed patterns for men on long-term AED use may be related to a health-seeking behaviour that differed from other men who were not on long-term AED.

The data granularity accessible from a large national database is one of the key strengths of this observational study. This is also the first study to investigate the association between AED usage and PCa risk in the Swedish population. Although PCa incidence can be ascertained from large cohorts such as PCBaSe, there are limitations to the wealth of information it entails. It was not possible to conclude that the indication for AED use was restricted to epilepsy since sodium valproate also has psychiatric indications [15].

The negative association observed between CCI and PCa in the results of this study may be explained by prostate-specific antigen (PSA) testing in men with comorbidities. Since PCa is a disease that is strongly related to PSA testing in asymptomatic men, men with comorbidities may be less likely to undergo PSA tests. The case-control design of this study also restricted the investigation of potential survival bias and other biases related to competing risks from death, as men lost to follow-up may not have survived long enough to develop cancer. Moreover, the focus of the current study was on AED usage in adults (follow-up up to 8.5 years); hence, it is limited in that it cannot examine the potential PCa long-term effects of childhood AED users.

## 5. Conclusion

Our results suggest a weak inverse association between AED exposure and risk of PCa. However, the results after adjustments for healthcare utilisation, analyses of cumulative doses of AED, and complementary analyses of individual AEDs with or without HDACi properties do not support that use of AED is associated with a meaningful reduction of the risk of PCa. Further research investigating the mechanistic properties of all AED is required to understand the effects of AED on the risk of PCa.

## Figures and Tables

**Table 1:** Characteristics of prostate cancer cases and their matched controls in PCBaSe.

Number of patients	Cases (*N* = 31,591)		Controls (*N* = 156,802)		OR	95% CI
Characteristics						
Age, *n* (%)						
≤50	326	(1.0)	1640	(1.0)	1.00	(ref.)
51–60	3654	(11.6)	18088	(11.5)	^*∗∗*^1.26	(0.72–2.20)
61–70	12579	(39.8)	62090	(39.6)	^*∗∗*^1.25	(0.69–2.26)
71–80	10966	(34.7)	54775	(34.9)	0.98	(0.53–1.79)
81+	4066	(12.9)	20209	(12.9)	1.01	(0.53–1.93)
Antiepileptic drugs use, *n* (%)						
No	29853	(94.5)	147128	(93.8)	1.00	(ref.)
Yes	1738	(5.5)	9674	(6.2)	0.89	(0.84–0.93)
Civil status, *n* (%)						
Single	11351	(35.9)	63001	(40.2)	1.00	(ref.)
Married	20240	(64.1)	93801	(59.8)	1.20	(1.17–1.23)
Education level^*∗*^, *n* (%)						
Low	9561	(30.3)	51353	(32.8)	1.00	(ref.)
Middle	13099	(41.5)	64304	(41.0)	1.10	(1.07–1.14)
High	8931	(28.3)	41145	(26.2)	1.18	(1.14–1.22)
CCI^*∗*^, *n* (%)						
0	25999	(82.3)	126396	(80.6)	1.00	(ref.)
1	2982	(9.4)	15666	(10.0)	0.92	(0.88–0.96)
2	1410	(4.5)	7311	(4.7)	0.93	(0.88–0.99)
3+	1200	(3.8)	7429	(4.7)	0.78	(0.73–0.83)
Outpatient visits 1–10 years prior to inclusion, *n* (%)						
No visits	12098	(38.3)	63160	(40.3)	1.00	(ref.)
1 visit	4662	(14.8)	21914	(14.0)	1.11	(1.07–1.16)
2 visits	3412	(10.8)	15965	(10.2)	1.12	(1.07–1.17)
3–5 visits	5615	(17.8)	25823	(16.5)	1.14	(1.10–1.18)
6–9 visits	2957	(9.4)	14063	(9.0)	1.10	(1.06–1.15)
10+ visits	2847	(9.0)	15877	(10.1)	0.94	(0.90–0.98)
Cumulative length of hospital stay 1–10 years prior to inclusion, *n* (%)						
No visits	19378	(61.3)	91725	(58.5)	1.00	(ref.)
1–3 days	4145	(13.1)	20111	(12.8)	0.97	(0.93–1.00)
4–7 days	3123	(9.9)	15233	(9.7)	0.96	(0.92–1.00)
1-2 weeks	2298	(7.3)	11970	(7.6)	0.89	(0.85–0.94)
3-4 weeks	1541	(4.9)	9159	(5.8)	0.78	(0.73–0.82)
4+ weeks	1106	(3.5)	8604	(5.5)	0.59	(0.56–0.63)

^*∗*^SD: standard deviation, education: low (elementary school), middle (9–12 years in education/gymnasium), and high (>12 years in education/university), and CCI: Charlson comorbidity index. ^*∗∗*^ Deviations from 1 are seen in the ORs because there is not a complete correspondence in age groups between cases and controls, as this would lead to a singular design matrix in the logistic regression model. Despite matching, ORs deviating from 1 with notable imprecision are seen due to controls being matched to cases based on year of birth, while age was calculated based on year and quarter of birth.

**Table 2:** Multivariable adjusted odds ratios (ORs) and 95% confidence intervals (CIs) by exposure of antiepileptic drug and cumulative defined daily dose overall and for subcohorts with low-risk, intermediate-risk, and high-risk or metastatic prostate cancer and their respective controls.

	Cases		Controls		Crude		Adjusted^a^		Adjusted^b^	
	*N*	(%)	*N*	(%)	OR	95% CI	OR	95% CI	OR	95% CI
Antiepileptic drug										
No	29853	(94.5)	147128	(93.8)	1.00	(ref.)	1.00	(ref.)	1.00	(ref.)
Yes	1738	(5.5)	9674	(6.2)	0.89	(0.84–0.93)	0.92	(0.87–0.97)	0.96	(0.91–1.02)
DDD of antiepileptic drug										
No drug	30345	(96.1)	149490	(95.3)	1.00	(ref.)	1.00	(ref.)	1.00	(ref.)
1–365 DDD	677	(2.1)	3710	(2.4)	0.90	(0.83–0.98)	0.93	(0.86–1.01)	0.98	(0.90–1.06)
1–5 year DDD	371	(1.2)	2219	(1.4)	0.82	(0.74–0.92)	0.86	(0.77–0.97)	0.93	(0.83–1.04)
6+ year DDD	198	(0.6)	1383	(0.9)	0.71	(0.61–0.82)	0.75	(0.64–0.87)	0.80	(0.68–0.93)
Low-risk or intermediate PCa										
Antiepileptic drug										
No	18157	(94.5)	89572	(93.9)	1.00	(ref.)	1.00	(ref.)	1.00	(ref.)
Yes	1065	(5.5)	5774	(6.1)	0.91	(0.85–0.97)	1.00	(0.94–1.07)	1.03	(0.96–1.10)

DDD of antiepileptic drug										
No drug	18481	(96.1)	90965	(95.4)	1.00	(ref.)	1.00	(ref.)	1.00	(ref.)
1–365 DDD	394	(2.0)	2181	(2.3)	0.89	(0.80–0.99)	0.98	(0.88–1.10)	1.00	(0.90–1.12)
1–5 year DDD	233	(1.2)	1327	(1.4)	0.86	(0.75–0.99)	0.99	(0.86–1.14)	1.03	(0.89–1.19)
6+ year DDD	114	(0.6)	873	(0.9)	0.64	(0.53–0.78)	0.73	(0.60–0.89)	0.75	(0.62–0.92)
High-risk or metastatic PCa										
Antiepileptic drug										
No	10924	(94.6)	53692	(93.6)	1.00	(ref.)	1.00	(ref.)	1.00	(ref.)
Yes	618	(5.4)	3643	(6.4)	0.83	(0.76–0.91)	0.84	(0.77–0.92)	0.89	(0.81–0.97)
DDD of antiepileptic drug										
No drug	11082	(96.0)	54594	(95.2)	1.00	(ref.)	1.00	(ref.)	1.00	(ref.)
1–365 DDD	252	(2.2)	1431	(2.5)	0.87	(0.76–0.99)	0.87	(0.76–1.00)	0.93	(0.81–1.06)
1–5 year DDD	127	(1.1)	830	(1.4)	0.75	(0.62–0.91)	0.76	(0.63–0.92)	0.82	(0.68–0.99)
6+ year DDD	81	(0.7)	480	(0.8)	0.83	(0.66–1.05)	0.84	(0.66–1.06)	0.90	(0.71–1.14)

^*∗*^DDD: defined daily dose; adjusted^a^: educational level, civil status, and Charlson comorbidity index (CCI); adjusted^b^: number of outpatient visits and cumulative length of hospital stay.

**Table 3:** Multivariable adjusted odds ratios (ORs) and 95% confidence intervals (CIs) for prostate cancer based on specific antiepileptic drug usage.

Antiepileptic drugs	Cases		Controls		Crude		Adjusted^a^		Adjusted^b^	
	*N*	(%)	*N*	(%)	OR	95% CI	OR	95% CI	OR	95% CI
Sodium valproate										
No	31474	(99.6)	156036	(99.5)	1.00	(ref.)	1.00	(ref.)	1.00	(ref.)
Yes	117	(0.4)	766	(0.5)	0.75	(0.62–0.92)	0.79	(0.65–0.96)	0.85	(0.70–1.04)
Carbamazepine										
No	31271	(99.0)	154852	(98.8)	1.00	(ref.)	1.00	(ref.)	1.00	(ref.)
Yes	320	(1.0)	1950	(1.2)	0.81	(0.72–0.92)	0.86	(0.77–0.97)	0.92	(0.81–1.03)
Lamotrigine										
No	31418	(99.5)	155872	(99.4)	1.00	(ref.)	1.00	(ref.)	1.00	(ref.)
Yes	173	(0.5)	930	(0.6)	0.92	(0.78–1.09)	0.95	(0.81–1.12)	1.00	(0.85–1.18)
Levetiracetam										
No	31498	(99.7)	156167	(99.6)	1.00	(ref.)	1.00	(ref.)	1.00	(ref.)
Yes	93	(0.3)	635	(0.4)	0.72	(0.58–0.90)	0.77	(0.62–0.96)	0.85	(0.69–1.06)
Oxcarbazepine										
No	31574	(99.9)	156730	(100.0)	1.00	(ref.)	1.00	(ref.)	1.00	(ref.)
Yes	17	(0.1)	72	(0.0)	1.17	(0.69–1.99)	1.20	(0.70–2.03)	1.28	(0.75–2.17)
Ethosuximide										
No	31591	(100.0)	156796	(100.0)						
Yes	0	(0.0)	6	(0.0)						
Topiramate										
No	31557	(99.9)	156638	(99.9)	1.00	(ref.)	1.00	(ref.)	1.00	(ref.)
Yes	34	(0.1)	164	(0.1)	1.03	(0.71–1.49)	1.07	(0.74–1.55)	1.12	(0.77–1.62)
Phenytoin										
No	31540	(99.8)	156471	(99.8)	1.00	(ref.)	1.00	(ref.)	1.00	(ref.)
Yes	51	(0.2)	331	(0.2)	0.76	(0.57–1.03)	0.81	(0.60–1.09)	0.85	(0.63–1.14)
Gabapentin										
No	30840	(97.6)	152958	(97.5)	1.00	(ref.)	1.00	(ref.)	1.00	(ref.)
Yes	751	(2.4)	3844	(2.5)	0.97	(0.90–1.05)	1.00	(0.92–1.08)	1.04	(0.96–1.13)
Pregabalin										
No	31047	(98.3)	153780	(98.1)	1.00	(ref.)	1.00	(ref.)	1.00	(ref.)
Yes	544	(1.7)	3022	(1.9)	0.89	(0.81–0.98)	0.92	(0.84–1.01)	0.97	(0.88–1.06)
Clonazepam										
No	31511	(99.7)	156310	(99.7)	1.00	(ref.)	1.00	(ref.)	1.00	(ref.)
Yes	80	(0.3)	492	(0.3)	0.81	(0.64–1.02)	0.85	(0.67–1.07)	0.91	(0.71–1.15)
Primidone										
No	31568	(99.9)	156719	(99.9)	1.00	(ref.)	1.00	(ref.)	1.00	(ref.)
Yes	23	(0.1)	83	(0.1)	1.38	(0.87–2.19)	1.42	(0.89–2.25)	1.42	(0.89–2.25)
Phenobarbital										
No	31577	(100.0)	156707	(99.9)	1.00	(ref.)	1.00	(ref.)	1.00	(ref.)
Yes	14	(0.0)	95	(0.1)	0.74	(0.42–1.29)	0.78	(0.44–1.37)	0.78	(0.45–1.38)
Lacosamide										
No	31586	(100.0)	156778	(100.0)	1.00	(ref.)	1.00	(ref.)	1.00	(ref.)
Yes	5	(0.0)	24	(0.0)	1.02	(0.39–2.69)	1.04	(0.40–2.72)	1.08	(0.41–2.85)
Zonisamide										
No	31589	(100.0)	156788	(100.0)	1.00	(ref.)	1.00	(ref.)	1.00	(ref.)
Yes	2	(0.0)	14	(0.0)	0.71	(0.16–3.14)	0.72	(0.16–3.18)	0.78	(0.18–3.44)
Eslicarbazepine										
No	31589	(100.0)	156801	(100.0)	1.00	(ref.)	1.00	(ref.)	1.00	(ref.)
Yes	2	(0.0)	1	(0.0)	10.00	(0.91–110)	9.39	(0.85–104)	8.50	(0.77–93.9)

^*∗*^adjusted^a^: educational level, civil status, and Charlson comorbidity index (CCI); adjusted^b^: for number of outpatient visits and cumulative length of hospital stay.

## Data Availability

The aggregated data used to support the findings of this study may be made available at a remote server upon application to the Prostate Cancer data Base Sweden reference group and the Research Ethics Authority, who can be contacted at par.stattin@surgsci.uu.se.

## Additional Points

Long-term treatment with antiepileptic drugs has been suggested to reduce the risk of prostate cancer due to their histone deacetylases inhibitory properties. Our study with over 188,000 men on antiepileptic drugs with comprehensive data on cancer characteristics addresses the need for more observational studies. Although our results elucidated an overall reduced risk of prostate cancer following antiepileptic drug use, we found no evidence for a stronger reduction related to the histone deacetylases inhibitory properties.
